# Involvement of the intestinal microbiota in the formation of neurodegenerative disorders

**DOI:** 10.1192/j.eurpsy.2023.1970

**Published:** 2023-07-19

**Authors:** A. Sidenkova

**Affiliations:** Psychiatry, Ural State Medical University, Yekaterinburg, Russian Federation

## Abstract

**Introduction:**

Increased life expectancy, increased prevalence of neurocognitive disorders, various aspects of the concept of “age” and pathogenic the influence of late age on the formation of cognitive deficit was the basis for this study. Bi-directional communication between the brain and the intestine is continuous and supported by the mechanisms that carry out the work of the axis “brain-gut”. Increased life expectancy, increased prevalence of neurocognitive disorders,various aspects of the concept of “age” and pathogenicthe influence of late age on the formation of cognitive deficit was the basis for this study. Bi-directional communicationbetween the brain and the intestine is continuous and supported by the mechanisms that carry out the work of the axis“brain-gut”

**Objectives:**

Studied relationships between microbiota of the gastrointestinal tract and CNS diverse and dynamic, including in relation to to age and the aging process. Studied relationships betweenmicrobiota of the gastrointestinal tract and CNSdiverse and dynamic, including in relation toto age and the aging process.

**Methods:**

Microbiotic a person’s profile is age-specific. Changes in microbial middle age increase mental, cognitive problems in the elderly and senile age.

**Results:**

Dysbiosis of the intestinal microbiota in AD triggers neuroinflammation, which contributes to the accumulation of Aβ in brain structures and pathological cleavage of the tau protein, which leads to disruption of the functions of microglia, hippocampus, and synaptic transmission. The emergence of a two-way connection through the vagus nerve system between the formations of the digestive tract containing microbiota and the CNS with the formation of a “vicious circle” with the development of age-related pathological processes in the CNS. The diverse and multilevel process of aging in its pathological form embraced the active participation of mental adaptation.

**Conclusions:**

Involvement of the microbiota in the pathogenesis of the disease Alzheimer’s suggests that the correction intestinal microflora may have potential value for the prevention of cognitive damage and / or be included in the therapeutic complex, which requires further study and analysis. Involvement of the microbiota in the pathogenesis of the diseaseAlzheimer’s suggests that the correctionintestinal microflora may have potentialvalue for the prevention of cognitive damage and / or be included in the therapeutic complex, whichrequires further study and analysis

**Disclosure of Interest:**

None Declared

